# Incarcerated Sternoclavicular Joint Intra-articular Disc Following Closed Reduction of a Posterior Dislocation Leading to Recurrent Anterior Instability

**DOI:** 10.7759/cureus.83027

**Published:** 2025-04-26

**Authors:** Graham Tytherleigh-Strong, Thomas J Melton

**Affiliations:** 1 Trauma & Orthopaedics, Addenbrooke's Hospital, Cambridge University Hospitals NHS Foundation Trust, Cambridge, GBR

**Keywords:** incarcerated disc, irreducible closed reduction, mri scan, sternoclavicular joint stabilisation, traumatic sternoclavicular joint dislocation

## Abstract

Traumatic posterior dislocations of the sternoclavicular joint (SCJ) are rare. One option for management is to undertake a closed reduction within 48 hours, confirmed by a post-reduction computed tomography (CT) scan.

We describe the case of a 17-year-old male patient who sustained a posterior SCJ dislocation that was treated within 48 hours by a closed reduction confirmed by CT. However, he went on to develop recurrent SCJ instability. A magnetic resonance imaging (MRI) arthrogram demonstrated that the intra-articular disc had been incarcerated at the back of the joint at the time of reduction and tears to both the anterior and posterior capsule. He underwent a successful open excision of the disc and hamstring tendon autograft reconstruction.

Following closed reduction for an acute posterior SCJ dislocation, we would recommend undertaking a post-reduction MRI scan to confirm the reduction and status of the soft tissues.

## Introduction

Following an acute traumatic posterior sternoclavicular joint (SCJ) dislocation, an attempted closed reduction, undertaken within 48 hours of the injury, has been recommended [1]. Due to the midline position of the sternum with the vertebral column directly behind, there are no plain imaging views that can reliably confirm a successful reduction. A computed tomography (CT) scan is considered the most appropriate check investigation [2]. However, previous studies have shown that there is a significant variation in symmetry between the SCJs in the normal population and suggested that a CT scan may be an unreliable investigation to assess joint instability [3].

We describe the case of a 17-year-old male patient who underwent what was considered a successful closed reduction following an acute traumatic posterior SCJ dislocation. This was confirmed by a post-reduction CT scan. However, he went on to develop recurrent SCJ instability. A magnetic resonance imaging (MRI) arthrogram demonstrated that the intra-articular disc had been incarcerated into the back of the joint at the time of reduction. He went on to undergo a successful open excision of the disc and hamstring tendon autograft reconstruction.

## Case presentation

A 17-year-old, right-handed, male, elite-level rugby player was referred to our unit with a history of recurrent left SCJ instability following a closed reduction. He had sustained a traumatic posterior SCJ dislocation playing rugby four months earlier and was seen the next day at his local hospital. A CT scan demonstrated a left posterior SCJ dislocation, and he underwent a closed reduction; this took five attempts. A post-reduction CT scan was considered to demonstrate that the joint was reduced. His arm was then immobilised in a sling for two weeks, and he was then advised to mobilise as pain permitted.

Following the relocation, he felt that his joint was never stable. He reported that the joint remained swollen and painful, with clicking and grinding on most movements. He was reluctant to fully protract or retract his shoulder due to apprehension.

At the initial review at our unit, his left SCJ was tender and swollen in comparison to the right side. There was palpable crepitus on protraction, retraction, and internal and external rotation of the joint. He was apprehensive about protraction and retraction, but his glenohumeral joint was intact. His Rockwood SCJ score was eight, the Quick Disabilities of the Arm, Shoulder, and Hand (DASH) score was 22.7, and the Oxford Shoulder Instability Score (OSIS) was 31 [4-6].

**Figure 1:**
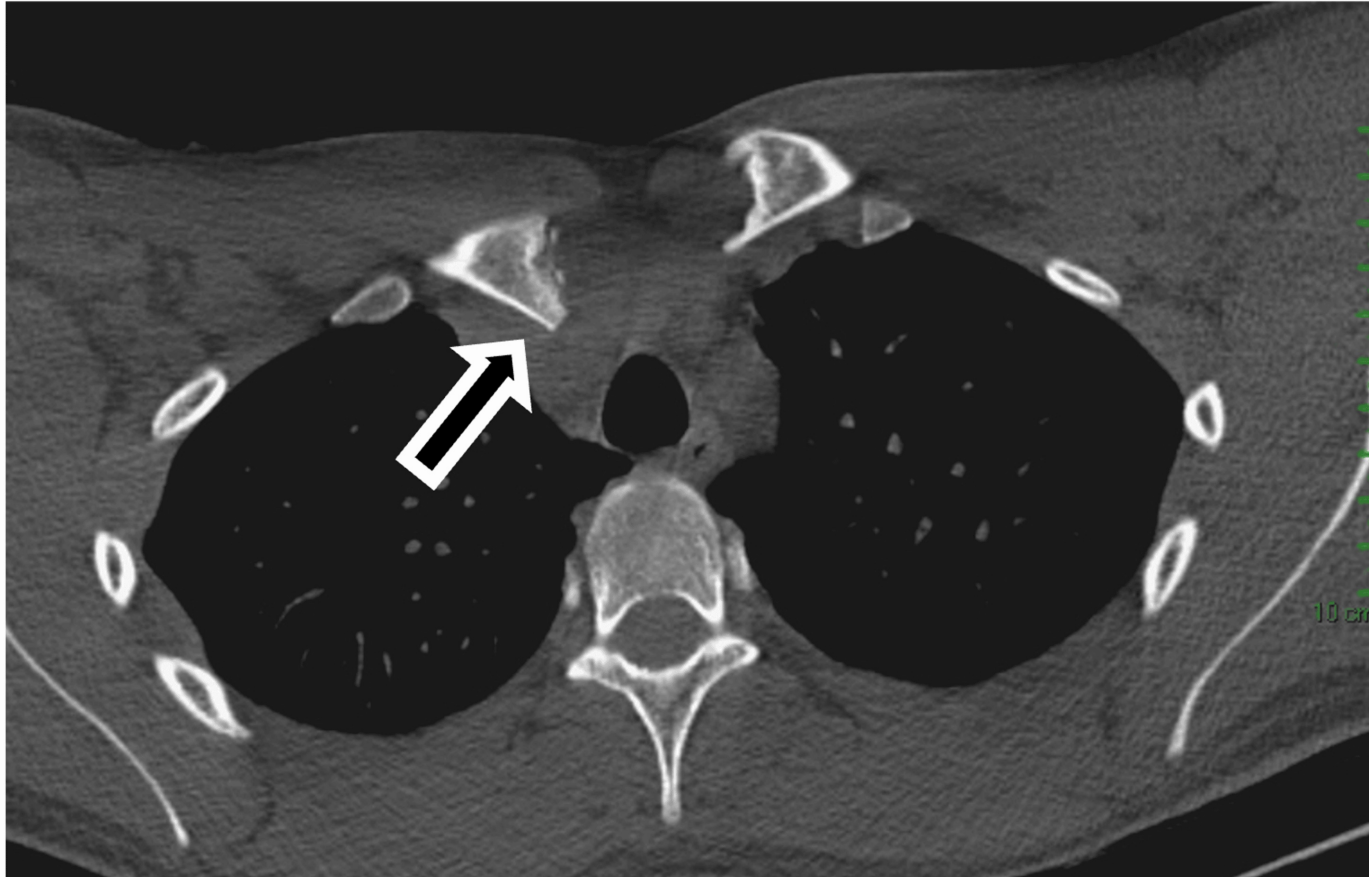
Posterior sternoclavciular joint dislocation Axial computed tomography scan of both sternoclavicular joints taken in the emergency department at initial admission. This demonstrates a posterior dislocation of the left sternoclavicular joint (black arrow).

**Figure 2:**
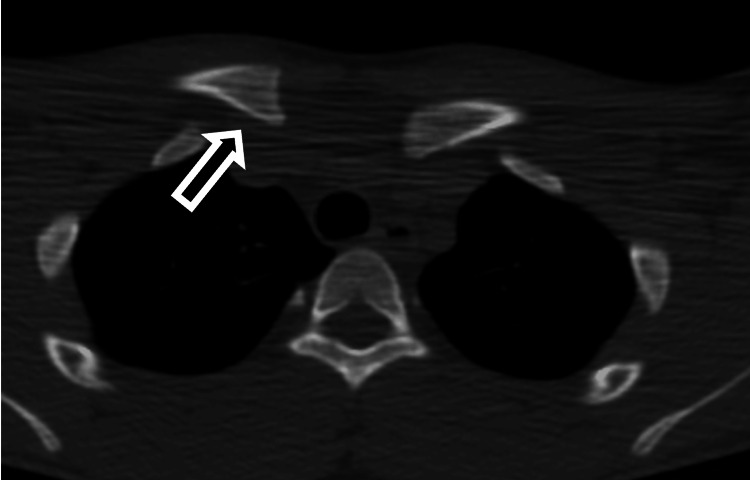
Post-reduction computed tomography scan Axial computed tomography scan of both sternoclavicular joints taken after closed reduction. The medial end of the left clavicle (black arrow) is positioned more anteriorly than the right.

His immediate post-injury CT scan showed a posterior dislocation of the left SCJ (Figure 1). The post-reduction CT scan showed that the medial end of the clavicle appeared to be in joint but was asymmetrically positioned in comparison to the right side, sitting more anteriorly (Figure 2).

**Figure 3:**
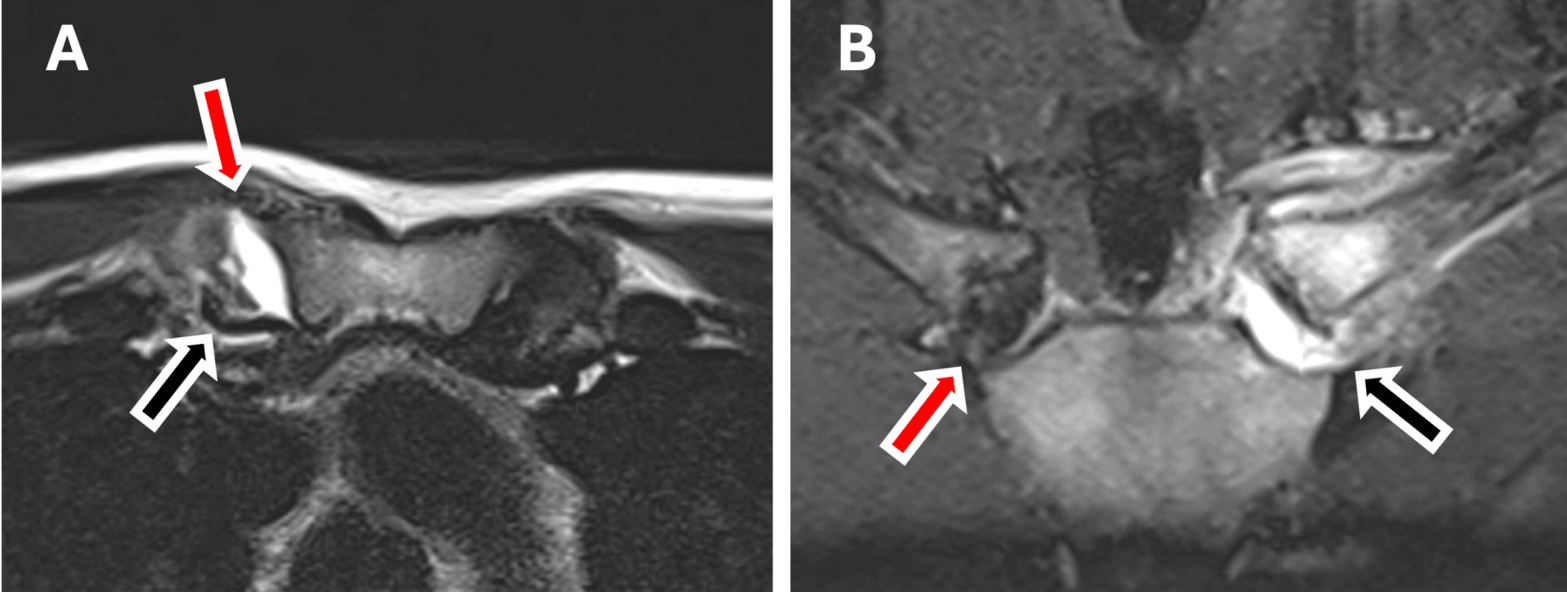
Magnetic resonance imaging scan A. Axial T2 magnetic resonance imaging arthrogram of both sternoclavicular joints. On the left, the gadolinium can be seen in the joint, penetrating through the anterior capsule (red arrow). The intra-articular disc lies posterior to the medial end of the clavicle, still attached to the sternum (black arrow); B. Coronal T2 magnetic resonance imaging arthrogram of both sternoclavicular joints. On the left, the joint space is filled with the gadolinium dye, and the disc is absent (black arrow). On the right, the intra-articular disc can be seen between the sternal and clavicular joint surfaces (red arrow).

An MRI arthrogram was ordered, as the injury was no longer acute, and demonstrated a joint effusion with anterior subluxation of the joint (Figure 3). There was a tear to both the anterior and posterior capsules, and the intra-articular disc was incarcerated behind the medial end of the clavicle.

A diagnosis of recurrent anterior and posterior SCJ instability due to an incarcerated disc was made. These findings were discussed with the patient and his parents, and further treatment options were considered. These were to either preserve his joint as it was, accepting that his symptoms were likely to continue, or to undergo an open excision of the intra-articular disc and a horizontal figure-of-eight reconstruction using a hamstring tendon autograft. The risks and benefits of the surgical procedure were explained, and the patient chose to undergo surgery.

**Figure 4:**
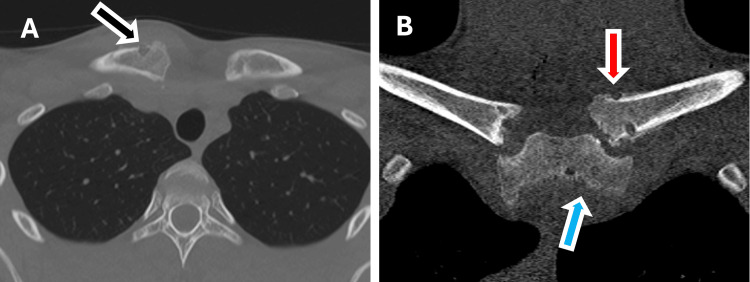
Post-operative computed tomography scan following removal of the disc and horizontal hamstring tendon autograft. A. Axial computed tomography scan demonstrating that the left clavicle is now symmetrically positioned with the right side. The superior anterior-to-posterior drill hole through the medial clavicle can be seen in the anterior cortex (black arrow). B. Coronal computed tomography scan demonstrating symmetry between the right and left clavicles. The two anterior-to-posterior drill holes can be seen in the medial clavicle (red arrow) and sternum (blue arrow).

Having obtained informed consent, the patient underwent an open excision of the intra-articular and a horizontal figure-of-eight hamstring tendon reconstruction using a previously described technique [7]. At surgery, the intra-articular disc was found to be positioned behind the medial end of the clavicle and still attached to the posterior capsule on the sternal side. The posterior capsule on the clavicular side of the joint was torn. The procedure was completed successfully. His post-operative CT scan showed that the joint was reduced and the drill holes were in the correct position (Figure 4).

Post-operatively, the patient’s arm was kept in a sling for two weeks, and he then underwent a standard rehabilitation programme without complication.

At the most recent follow-up, 18 months after the procedure, the patient considered that his left SCJ was back to normal. He had returned to playing elite-level rugby; his Rockwood SCJ score was 15, Quick-DASH score was 0, OSIS score was 48, and Single Assessment Numeric Evaluation (SANE) score was 100 [8].

## Discussion

Traumatic posterior dislocations of the SCJ are rare and are usually the result of a high-energy injury [9]. Given this rarity, the number of reported cases in the literature is relatively sparse, made up of case reports and small case series, and tends to focus on open reduction and surgical stabilisation. As a result, information on the success and outcome of patients treated with a closed reduction is very limited.

An attempted closed reduction within 48 hours of injury, in the first instance, has been recommended. It is generally now considered that this should be undertaken in an operating theatre, the patient under general anaesthetic and with consultation with a vascular or thoracic surgeon [10, 11]. Two main techniques have been described to reduce the posteriorly dislocated clavicle; one is using a towel clip to hold the clavicle and pulling it anteriorly. The other is the abduction traction technique, where the shoulder is abducted to 90° and traction is applied, followed by an extension force resulting in anterior translation of the medial clavicle back into the joint [12]. Following reduction, a check CT scan is recommended [10].

In a case series of 30 posterior SCJ dislocations and Salter-Harris type 2 fracture dislocations, 17 patients had a posterior SCJ dislocation [13]. Fourteen were treated within three weeks, of which nine underwent an initial closed reduction. Four (44%) of these required a subsequent open reduction and stabilisation because of failure of reduction or persistent instability. One of these patients developed anterior instability 12 days after closed reduction. Of the 12 patients who underwent an immediate open reduction, six had a significant intra-articular disc tear that required resection. In another series of 21 patients, all of the patients underwent an initial closed reduction, of which 13 (61.9%) were deemed unsuccessful and required open reduction and stabilisation [14]. However, only four of these patients were treated within three weeks of their initial injury.

There are two main reasons why an attempted closed reduction of a posterior SCJ dislocation may fail. The first is a failure of technique, and the second is that the torn posterior capsule and disc, which is always torn off of the medial end of the clavicle, are trapped between the sternal articular notch and the clavicle, preventing reduction. In the reported case, we presume that, at the time of initial closed reduction, the posterior capsule and disc were caught between the sternal notch and clavicle. We suspect that over the course of the five attempts at reduction, the capsule and disc were sequentially crushed until the medial end of the clavicle was forced past the disc, flicking it posteriorly behind the clavicle. As a result, with the anterior capsule also torn, the clavicle ended up being subluxed anteriorly.

At the advent of CT scanning, a case series of posteriorly and superiorly dislocated SCJs diagnosed by a CT scan demonstrated how easily they could diagnose dislocations [15]. A logical assumption was made that, in reverse, a CT scan could reliably confirm a successful reduction. However, numerous subsequent studies found that there was a significant variation in symmetry between the SCJs in the normal population and suggested that this was unreliable. As a result, and as evidenced by this case, unless a CT scan of the position of both SCJs prior to dislocation is available, a post-reduction CT scan following dislocation may not be reliable.

Unlike most other joints (shoulder, knee, and ankle), no radiological assessment of the stabilising soft tissue structures of the SCJ, in the form of an MRI scan, is routinely undertaken. Previous SCJ studies have described the use of MRI scans for the diagnosis of disc tears and following injury, but not for the confirmation of a successful reduction [16-18].

For the reported case, particularly considering the difficulty in the reduction and the patient’s immediate concern of ongoing instability, undertaking an immediate MRI scan may have been indicated. In the acute phase, due to the associated haemarthrosis, an MRI arthrogram would not usually be indicated.

## Conclusions

Following closed reduction for an acute posterior SCJ dislocation, incarceration of the capsular soft tissues can lead to ongoing instability. In addition to a post-reduction CT scan, we recommend undertaking a post-reduction MRI scan to assess the status of the soft tissues.

## References

[1] Sernandez H, Riehl J (2019). Sternoclavicular joint dislocation: a systematic review and meta-analysis. J Orthop Trauma.

[2] Logan C, Shahien A, Altintas B, Millett PJ (2018). Rehabilitation following sternoclavicular joint reconstruction for persistent instability. Int J Sports Phys Ther.

[3] Tuscano D, Banerjee S, Terk MR (2009). Variations in normal sternoclavicular joints; a retrospective study to quantify SCJ asymmetry. Skeletal Radiol.

[4] Rockwood CA Jr, Groh GI, Wirth MA, Grassi FA (1997). Resection arthroplasty of the sternoclavicular joint. J Bone Joint Surg Am.

[5] Gummesson C, Ward MM, Atroshi I (2006). The shortened disabilities of the arm, shoulder and hand questionnaire (QuickDASH): validity and reliability based on responses within the full-length DASH. BMC Musculoskelet Disord.

[6] Dawson J, Fitzpatrick R, Carr A (1999). The assessment of shoulder instability. The development and validation of a questionnaire. J Bone Joint Surg Br.

[7] Tytherleigh-Strong G, Sabharwal S, Peryt A (2022). Clinical outcomes and return to sports after open reduction and hamstring tendon autograft reconstruction in patients with acute traumatic first-time posterior dislocation of the sternoclavicular joint. Am J Sports Med.

[8] Williams GN, Gangel TJ, Arciero RA, Uhorchak JM, Taylor DC (1999). Comparison of the single assessment numeric evaluation method and two shoulder rating scales. Outcomes measures after shoulder surgery. Am J Sports Med.

[9] Nettles JL, Linscheid RL (1968). Sternoclavicular dislocations. J Trauma.

[10] Iwai T, Tanaka K, Okubo M (2018). Closed reduction of a posterior sternoclavicular joint dislocation: a case report. Trauma Case Rep.

[11] Tepolt F, Carry PM, Heyn PC, Miller NH (2014). Posterior sternoclavicular joint injuries in the adolescent population: a meta-analysis. Am J Sports Med.

[12] Buckerfield CT, Castle ME (1984). Acute traumatic retrosternal dislocation of the clavicle. J Bone Joint Surg Am.

[13] Laffosse JM, Espié A, Bonnevialle N (2010). Posterior dislocation of the sternoclavicular joint and epiphyseal disruption of the medial clavicle with posterior displacement in sports participants. J Bone Joint Surg Br.

[14] Groh GI, Wirth MA, Rockwood CA Jr (2011). Treatment of traumatic posterior sternoclavicular dislocations. J Shoulder Elbow Surg.

[15] Levinsohn EM, Bunnell WP, Yuan HA (1979). Computed tomography in the diagnosis of dislocations of the sternoclavicular joint. Clin Orthop Relat Res.

[16] Benitez CL, Mintz DN, Potter HG (2004). MR imaging of the sternoclavicular joint following trauma. Clin Imaging.

[17] Tytherleigh-Strong G, Rashid A, Lawrence C, Morrissey D (2017). Arthroscopic sternoclavicular joint diskectomy for acute and chronic tears. Arthroscopy.

[18] Tytherleigh-Strong GM, Getgood AJ, Griffiths DE (2012). Arthroscopic intra-articular disk excision of the sternoclavicular joint. Am J Sports Med.

